# Difference in Cd accumulation among varieties with different growth duration corresponding to typical agro-climate condition in rice ratooning system

**DOI:** 10.3389/fpls.2024.1383428

**Published:** 2024-05-08

**Authors:** Shuai Yuan, Yanfang Jiang, Pingping Chen, Naimei Tu, Wenxin Zhou, Zhenxie Yi

**Affiliations:** College of Agronomy, Hunan Agricultural University, Changsha, China

**Keywords:** growth duration, dry matter accumulation, cadmium concentration and accumulation, ratoon rice, temperature

## Abstract

**Introduction:**

The ratoon rice planting area is gradually expanding, but there has been relatively little research on ratoon rice grains contaminated with Cd.

**Methods:**

In this study, five ratoon rice varieties were selected and divided into three groups according to early-maturity (growth duration: 100–110 days), mid-maturity (growth duration: 110–120 days) and late-maturity (growth duration: 120–130 days) varieties. Field experiments were done to study the differences in Cd accumulation among ratoon rice varieties with different growth duration.

**Results:**

The results showed that the Cd accumulation and concentration of grains spikelet at each growth stage in the main crop were in the order of late-maturity > mid-maturity > early-maturity varieties. However, the trends in Cd concentration and accumulation in grains spikelet during the ratoon crop were the opposite. Analysis found that as the growth duration of the variety extended, the accumulated temperature and daily average temperature in the main crop increased, which significantly increased the translocation factors of Cd from root, stem, and leaf to grains spikelet, and increased the daily average Cd accumulation rate in grains spikelet. The daily average temperature in the ratoon crop increased as the growth duration shortened. The early-maturity variety had higher Cd accumulation in stubble, which promoted the translocation of Cd from the root, stem, and leaf of the plant to the grains spikelet.

**Discussion:**

Therefore, appropriately shortening the growth duration of the main crop and extending the growth duration of the ratoon crop are important ways to reduce Cd accumulation in ratoon rice in areas with mild Cd pollution.

## 1 Introduction

Rice (Oryza sativa L.) is one of the most important food crops in the world and more than 50% of the world’s population depend on rice as their staple food (Lin et al., 2019). Ratoon rice has a long history of cultivation as an important rice farming model. Ratoon rice production exploits rice regeneration characteristics. More specifically, certain cultivation and management measures are adopted to induce the germination of the dormant buds on the main rice crop stubble, which is followed by heading, flowering, grain filling and maturity (Zhang et al., 2021). However, in the past, the development momentum has been sluggish for a long time owing to the low yield of ratoon rice. In recent years, rice production costs have increased, and economic benefits have decreased. This has caused a widespread move from double to single cropping in double-cropping rice areas, resulting in a substantial waste of land, temperature and light resources, and affecting food security (Singh et al., 2016). Because ratoon rice has the advantages of saving costs, increasing income, increasing yield and reducing labor intensity, people have begun to focus on ratoon rice again. Owing to the selection of high-yield and high-quality varieties of ratoon rice, the optimization of nutrient and water management, research and development of comprehensive pest and disease prevention and control technology, and the matching of agricultural machinery and agronomy, the yield of ratoon rice has been greatly improved (Huang et al., 2022; He et al., 2023). The long grain filling time during the ratoon crop of ratoon rice allows sufficient accumulation of photosynthetic products, which is conducive to the formation of good rice quality (Li et al., 1990). The amount of pesticides used in the production of ratoon rice is lower than that of single cropping rice and double-cropping rice; thus, the risk of pesticide residues in ratoon rice is significantly reduced (Yuan et al., 2022). However, current research on the heavy metal accumulation characteristics of ratoon rice is still very limited.

With rapid industrial and socioeconomic development, the problems of heavy metal pollution in cultivated soil and excessive heavy metals in agricultural products have become increasingly prominent. Cadmium (Cd) is a major heavy metal that is relatively widely dispersed because of agricultural, mining, and other industrial activities as well as vehicle exhaust emissions. Research has shown that the exceedance rate of soil pollutants in China’s farmland was 19.4%, of which 7.0% was Cd (Hu et al., 2016). Cd enters the human body through the food chain. Once it exceeds the human body’s tolerance level, it will seriously harm the kidneys and bones (Jin et al., 2021). Rice is one of the food crops most likely to accumulate Cd. Therefore, for countries where rice is the staple food, reducing Cd in rice is of great practical significance to protect consumer health. Previous studies have shown that soil conditions and cultivation practices (water, fertilizer, density) have a significant impact on Cd accumulation in rice (Hu et al., 2013; Yong et al., 2017; Zhang et al., 2023). In addition, the Cd concentration of rice varies among different varieties. However, research on differences in the Cd concentration among varieties has focused on rice subspecies (indica and japonica), production season (early, mid, and late rice), and different genotypes of the same type of rice (Xin-Lu et al., 2017; Mei-rui et al., 2020; Ran et al., 2021).

The growth duration of rice varieties differs, thus the climatic factors happening during the growth duration of each ratoon are different. At the same time, the accumulation and translocation of Cd in rice are closely related to climatic factors (such as rainfall, temperature). Research shows that temperature has a significant impact on the translocation and accumulation of Cd from the mid-filling stage to maturity stage of rice (Pengju et al., 2018). Therefore, the different growth cycles of different rice varieties may be one of the influencing factors leading to differences in Cd accumulation in rice. At present, comparative research on Cd accumulation among rice varieties mainly focus on the differences in genetic characteristics of the varieties themselves (Chen et al., 2023), and there is no research on the impact of different growth duration of rice on Cd accumulation. At the same time, most of the studies on Cd accumulation comparisons among varieties are single-cropping rice and double-cropping rice (Shin et al., 2021), and there are few studies on ratooning rice varieties.

This study selected five ratoon rice varieties and divided them into three groups according to early-maturity (Xiangzaoxian 45), mid-maturity (Y liangyou 911, Hengliangyou Jinnonngsimiao), and late-maturity (Yongyou 4149, Yliangyou 9918) varieties. Field experiments were conducted to study the differences in Cd accumulation and translocation among ratoon rice varieties with different growth duration, and to explore the mechanism of the differences. The main purposes of this study were: (1) To compare the differences in Cd concentration and accumulation among ratoon rice varieties with different growth duration; (2) to study the impact mechanism of different growth duration, combined with meteorological data, on Cd accumulation and translocation in ratoon rice; (3) to explore appropriate Cd reduction methods based on the dynamic distribution of Cd accumulation in the main and ratoon crops of ratoon rice varieties with different growth duration.

## 2 Materials and methods

### 2.1 Site and materials

This study was conducted in a paddy field mildly contaminated with Cd in Hengyang county (26°97′ N, 111°37′ E), Hunan province, China in 2021. A typical rice-based double-cropping system was used in the study area, which is located in central China and has a subtropical monsoon climate with an annual precipitation of 1,411 mm and a mean annual temperature of 18.3°C. The study site contains red soil with the following characteristics: bio-available Cd concentration, 0.17 mg kg^−1^; total Cd concentration, 0.47 mg kg^−1^; pH, 6.01; cation exchange capacity, 7.26 c mol kg^−1^; alkaline hydrolyzable nitrogen concentration, 158.23 mg kg^−1^; available phosphorus concentration, 33.21 mg kg^−1^; available potassium concentration, 48.98 mg kg^−1^; organic matter concentration, 24.53 mg kg^−1^; total nitrogen concentration, 1.41 mg g^−1^; total phosphorus concentration, 0.59 mg g^−1^; and total potassium concentration, 9.45 mg g^−1^. A total of five ratoon rice varieties were tested, which were divided into three groups according to different growth duration: early-maturity variety-conventional *indica* rice Xiangzaoxian 45; mid-maturity variety-*indica* hybrid rice Hengliangyou jinnongsimiao, *indica* hybrid rice Y Liangyou 911; and late-maturity variety-*indica* hybrid rice Y Liangyou 9918, *indica–japonica* hybrid rice Yongyou 4149. The specific situation of the growth duration grouping of each ratoon rice variety is shown in **Table 1**.

**Table 1:** Ratoon rice varieties and their growth duration.

Variety type	Variety name	Variety code	Growth duration grouping (day)	The growth duration in this experiment (day)
Early-maturity variety	Xiangzaoxian 45	XZX-45	110	107
Medium-maturity variety	Y Liangyou 911	YLY-911	120	120
	Hengliangyou Jinnonngsimiao	HLY		120
Late-maturity variety	Yongyou 4149	YY-4149	130	131
	Y Liangyou 9918	YLY-9918		131

This experiment used a single-factor randomized block experiment, with each cultivar analyzed in triplicate (Liu et al., 2013). The study plots (40 m^2^) were separated from the surrounding area using protective rows (0.3 m width × 0.3 m height) covered with polyethylene film. Seeds for all five cultivars were sown on March 20 and seedlings were transplanted on April 26. The spacing among plants and rows was 20 cm × 20 cm, with 2-3 seedlings (hybrid rice) or 4–5 seedlings (conventional rice) per hill. Independent irrigation and drainage outlets were installed. Water management practices were the same for each plot to exclude the effects of water on the soil Cd concentration. The stubble height when the main crop was harvested was approximately 35 cm. Other management practices were consistent with conventional local practices.

### 2.2 Measurements and methods

Soil samples (0-20 cm depth) were collected using a five-point sampling method before seedlings were transplanted. After air-drying the soil samples, they were ground and passed through 20- and 100-mesh sieves. The soil pH was determined by adding CO_2_-free distilled water to the soil (water:soil ratio of 2.5:1) and then analyzing the extract using a PHSJ-3FX pH meter (Shanghai INESA Scientific Instrument Co., Ltd. Shanghai, China). The total Cd concentration of each soil sample was determined after adding 0.5 g dry soil to a mixture comprising HF, HClO_4_ and HNO_3_ in a DS-360 graphite digestion box (China National Analytical Center, Guangzhou, China). The bioavailable Cd concentration was determined after incubating a mixture comprising 5 g dry soil and 0.1 mol CaCl_2_ (soil:liquid ratio of 1:10) at 25°C for 2 h with mixing (250 rpm). The total Cd and CaCl_2_-extracted Cd concentrations in the solutions were determined using an AA800 graphite furnace atomic absorption spectrometer (PerkinElmer, Waltham, MA, USA). A volumetric method previously used for agricultural soil chemical analyses was used to determine the organic matter and alkaline nitrogen concentrations of the soil samples. The available phosphorus concentration was measured using a colorimetric method. The available potassium concentration was determined using a flame photometer. The total nitrogen, total phosphorus, and total potassium concentrations were determined according to a semi-micro Kjeldahl method.

Samples were taken at the full-heading stage, milky stage and maturity stage in both the main and ratoon crops. The corresponding month and date of each growth stage in the main and ratoon crops of different ratoon rice varieties are shown in **Table 2**. The yearly temperature fluctuation (January to December) for last 5 years in **Supplementary Figure S1**. The climatic condition data corresponding to each growth stage of the main and ratoon crops of different ratoon rice varieties are shown in **Table 3**. Collect daily surface meteorological data in Hengyang County, Hengyang City, Hunan Province, China from March to October 2021, including rainfall, average temperature, maximum temperature, minimum temperature and other data. The data comes from the National Meteorological Science Data Center (http://data.cma.cn). For some missing data, the average value of the meteorological element in the adjacent 2 days on that day is used as a replacement.

**Table 2:** The time of growth stage in the main and ratoon crops of ratoon rice varieties at different growth duration .

Season	Variety	Before the Full-Heading stage (M-D)	Full-Heading stage to Milky stage (M-D)	Milky stage to Maturity stage (M-D)	Full growth duration (day)
Main crop	XZX-45	03.20 -06.11	06.12 - 06.21	06.22-07.04	107
	YLY-911	03.20 -06.15	06.16 -06.29	06.30 - 07.17	120
	HLY				
	YY-4149	03.20 -0 6.19	06.20- 07.06	07.07 - 07.28	131
	YLY-9918				
Ratoon crop	XZX-45	07.05 - 08.16	08.17 - 8.27	08.28 - 09.11	69
	YLY-911	07.18 - 08.31	09.01 - 09.14	09.15 - 10.03	78
	HLY				
	YY-4149	07.29 - 09.14	09.15 -10.02	10.03 - 10.25	89
	YLY-9918				

XZX-45, Xiangzaoxian 45; YLY-911, Y Liangyou 911; HLY, Hengliangyou Jinnonngsimiao; YY-4149, Yongyou 4149; YLY-9918, Y Liangyou 9918. M-D, month-date.

**Table 3:** Climate changes during the main and ratoon crop of ratoon rice varieties at different growth duration.

Season	Growth stage	Variety	Daily average temperature (°C)	Daily maximum temperature (°C)	Daily minimum temperature (°C)	Rainfall (mm)	Active accumulated temperature (°C)	Effective accumulated temperature (°C)
Main crop	Sowing stage to Full heading stage	XZX-45	21.80	26.43	18.37	355.6	1806.7	996.7
		YLY-911	22.24	26.86	18.82	355.6	1933.2	1083.2
		HLY						
		YY-4149	22.58	27.23	19.16	375.2	2053	1163
		YLY-9918						
	Full heading stage to Milky stage	XZX-45	30.64	35.55	27.21	43.1	306.4	206.4
		YLY-911	29.49	34.03	26.34	133.80	412.8	272.8
		HLY						
		YY-4149	29.53	33.85	26.42	146.2	502.0	332.0
		YLY-9918						
	Milky stage to Maturity stage	XZX-45	29.15	33.34	26.17	122.7	378.9	248.9
		YLY-911	30.52	34.89	27.17	39.6	549.3	369.3
		HLY						
		YY-4149	30.89	35.47	27.36	43.7	679.6	459.6
		YLY-9918						
Ratoon crop	Before the Full heading stage	XZX-45	31.00	35.94	27.40	60.5	1332.8	902.8
		YLY-911	30.90	35.77	27.07	67.9	1390.3	940.3
		HLY						
		YY-4149	29.64	34.45	26.04	68.8	1422.7	942.7
		YLY-9918						
	Full heading stage to Milky stage	XZX-45	30.51	35.98	26.43	13.1	335.6	225.6
		YLY-911	26.55	30.54	23.71	30.0	371.7	231.7
		HLY						
		YY-4149	21.88	24.93	19.97	117.4	393.8	213.8
		YLY-9918						
	Milky stage to Maturity stage	XZX-45	27.98	32.41	24.57	31.8	419.7	269.7
		YLY-911	21.99	25.09	19.92	149.3	417.8	227.8
		HLY						
		YY-4149	18.18	21.45	15.70	65.1	418.2	188.2
		YLY-9918						

XZX-45, Xiangzaoxian 45; YLY-911, Y Liangyou 911; HLY, Hengliangyou Jinnonngsimiao; YY-4149, Yongyou 4149; YLY-9918, Y Liangyou 9918.

Before sampling, record the number of tillers of 50 rice plants in each plot, and then take 5 rice plants from each plot based on the average number of tillers per plant. Each plant was washed and then the roots were immersed in 0.1 mol L^-1^ hydrochloric acid for 15 min to remove Cd adsorbed to the root surface. The roots were subsequently washed three times with tap water, rinsed three times with deionized water, and dried. The main crop samples were divided into the stem, leaf, and panicle, with the panicle further divided into the branches, empty grains and grains spikelets. For the ratoon crop, the regenerated tillers were divided according to their node position (i.e., regenerated tillers of the second, third, and fourth or fifth node from the top). The tillers at the same node position were divided into the stem, stubble, leaf, and panicle, with the panicle further divided into the branches, empty grains and grains spikelets. All samples were bagged and packaged separately and were incubated in an oven at 105°C for 0.5 h and then dried to a constant weight at 80°C. Then they were crushed separately using a stainless steel plant sample crusher (Baijie 304, Suzhou, China). For Cd concentration, 0.5g of plant samples were weighed and digested with a mixed acid solution (HF-HClO_4_-HNO_3_) in a graphite digestion box (DS-360; China National Analytical Center, Guangzhou, China). Cd concentration in various plant parts was determined by graphite furnace atomic absorption spectrometry (AA800; Perkin Elmer, USA).

### 2.3 Calculating the Cd translocation and bio-accumulation factors

The Cd translocation factor (TF) for the rice plants was calculated using the following formula based on the Cd concentration of the upper tissues (Cd_m_) and the Cd concentration of the lower tissues (Cd_n_) (Equations 1–4):


(1) $$TF_{n}{\;}_{-to-m}=\;Cd_{m}/Cd_{n}$$

(2) $$Cd\;accumulation\;\left(mg\;ha^{-1}\right)\;=\;Cd\;concentration\;\times \;dry\;matter\;weight$$

The Cd accumulation among consecutive growth stages B and A was calculated as follows:


(3) $$Cdaccumulation_{A\;to\;B}\;(mgha^{-1})=\;Cdaccumulation_{B}-Cdaccumulation_{A}$$

(4) $$Average\;daily\;Cd\;accumulation\;rate\;\left(mg\;ha^{-1}d\right))\;=\;Cd\;accumulation/number\;of\;days\;in\;the\;growth\;stage$$

### 2.4 Statistical analysis

One-way analyses of variance for the different treatments were performed using the SPSS 24 software (IBM, Armonk, NY, USA). Graphs were drawn using the Origin 2021 program (OriginLab, Northampton, MA, USA).

## 3 Results

### 3.1 Differences in dry matter weight of ratoon rice varieties with different growth duration

**Figure 1:**
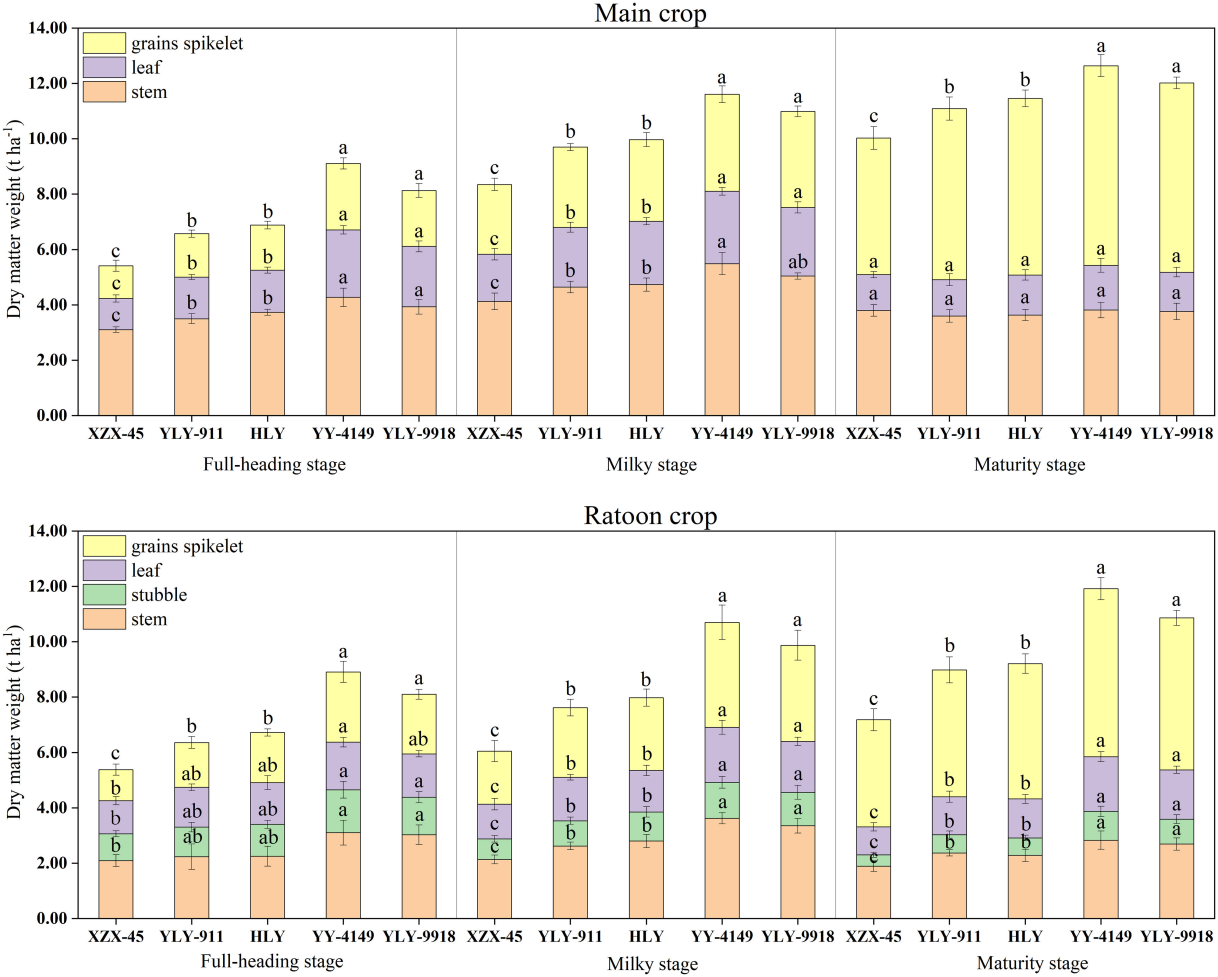
Dynamic changes in dry matter accumulation in the main and ratoon crops of ratoon rice varieties with different growth duration (t ha^-1^). Early-maturity variety: XZX-45, Xiangzaoxian 45; mid-maturity variety: YLY-911, Y Liangyou 911; HLY, Hengliangyou Jinnonngsimiao; late-maturity variety: YY-4149, Yongyou 4149; YLY-9918, Y Liangyou 9918. Significant differences (P< 0.05) among cultivars were determined by least significant difference tests and are indicated by different lowercase letters.

As shown in **Figure 1**, there were significant differences in dry matter at each growth stage among ratoon rice varieties with different growth duration. For the main crop, the dry matter weight of each organ at the full-heading stage and milky stage was higher in the late-maturity varieties (YLY-9918, YY-4149), followed by the mid-maturity varieties (HLY, YLY-911), and then the early-maturity variety (XZX-45). The differences among varieties with different growth duration was significant. At the maturity stage, there was no significant difference in the dry matter weight of stems and leaves among varieties with different growth duration. The dry matter weight of grains spikelet was in the order of late-maturity > mid-maturity > early-maturity varieties, and the difference was significant. During the ratoon crop, the dry matter weight of each organ at each growth stage among varieties with different growth duration showed the trend of late-maturity > mid-maturity > early-maturity varieties, and the differences among varieties with different growth duration in the milky stage and maturity stage was significant.

### 3.2 Differences in Cd concentration among ratoon rice varieties with different growth duration

**Figure 2:**
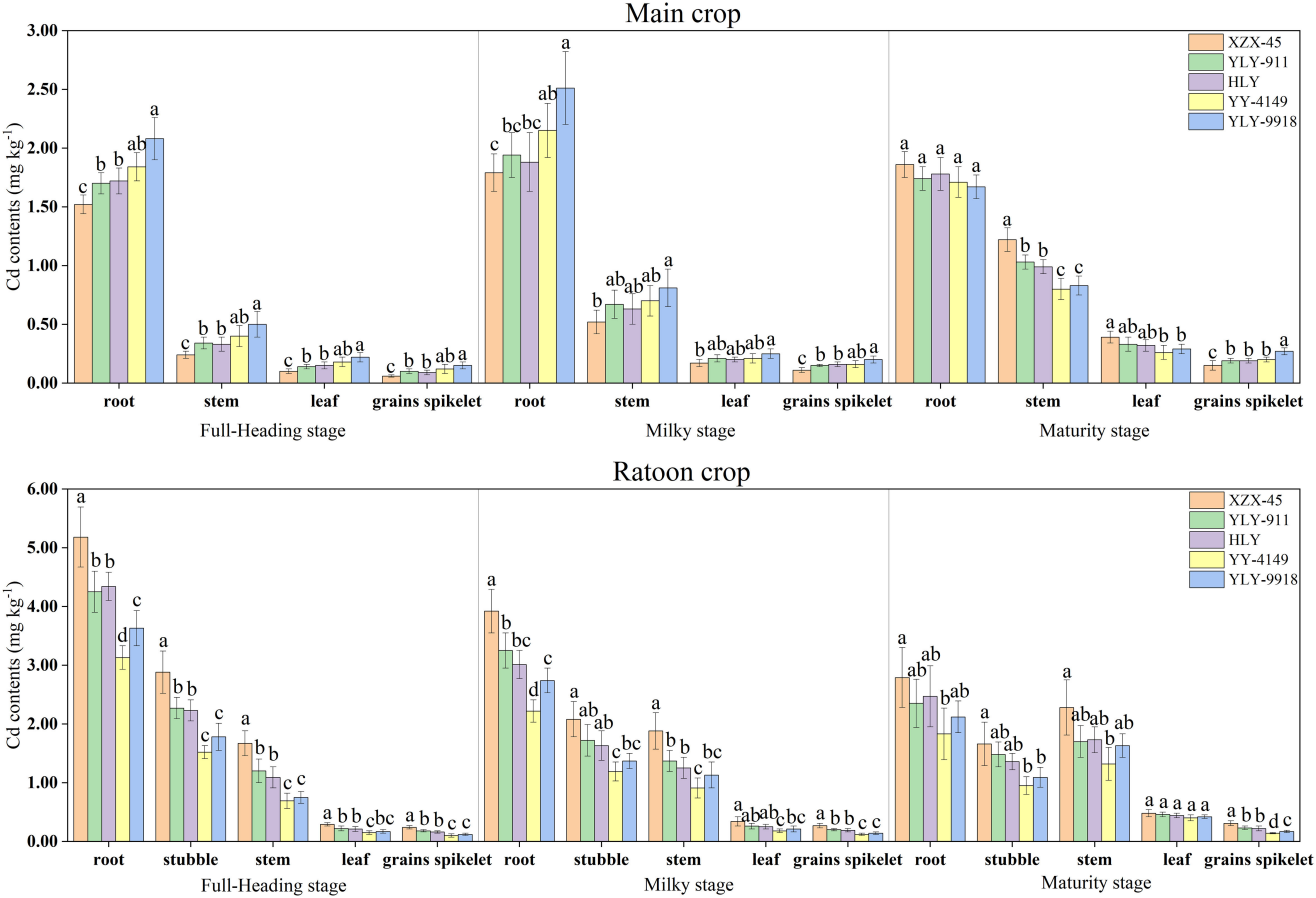
Cadmium (Cd) concentration at each growth stage in the main and ratoon crops of ratoon rice varieties with different growth duration (mg kg^-1^). Early-maturity variety: XZX-45, Xiangzaoxian 45; mid-maturity variety: YLY-911, Y Liangyou 911; HLY, Hengliangyou Jinnonngsimiao; late-maturity variety: YY-4149, Yongyou 4149; YLY-9918, Y Liangyou 9918. Significant differences (P< 0.05) among cultivars were determined by least significant difference tests and are indicated by different lowercase letters.

As shown in **Figure 2**, the Cd concentration in the organs of ratoon rice varieties with different growth duration differed significantly. In the main crop, as the growth stage progressed, the Cd concentration in the root of each variety first increased and then decreased, while the Cd concentration in the stem, leaf and grains spikelet showed an increasing trend. The Cd concentration in each organ at the full-heading stage and milky stage showed the trend of late-maturity > mid-maturity > early maturity varieties. At the maturity stage, there was no significant difference in root Cd concentration among varieties with different growth duration, while the Cd concentration in the stem and leaf was in the order of early-maturity > mid-maturity > late-maturity varieties. The difference among early-maturity and late-maturity varieties was significant. The Cd concentration in grains spikelet showed the opposite pattern, in the order of late-maturity > mid-maturity > early-maturity varieties, among which the late-maturity variety Y Liangyou 9918 was significantly higher than other varieties. In the ratoon crop, as the growth stage progressed, the Cd concentration in the root and stubble of each variety showed a decreasing trend, while the Cd concentration in the stem, leaf and grains spikelet showed an increasing trend. The Cd concentration in each organ at each growth stage among varieties in the ratoon crop was in the order of early maturity > mid-maturity > late maturity varieties. For grains spikelet, the difference in Cd concentration among varieties with different growth duration was significant.

### 3.3 Differences in Cd accumulation among ratoon rice varieties with different growth duration

As shown in **Table 4**, there were significant differences in organ Cd accumulation at each growth stage in the main crop among ratoon rice varieties with different growth duration. At the transplanting to full-heading stage and the full-heading to milky stage, the Cd accumulation in each organ was in the order of late-maturity > mid-maturity > early-maturity varieties, and the differences among varieties with different growth duration were significant. In the milky to maturity stage, Cd accumulation in the stem and leaf showed the trend of early-maturity > mid-maturity > late-maturity varieties, while the Cd accumulation in grains spikelet showed the opposite pattern, with late-maturity > mid-maturity > early-maturity varieties. The differences among varieties with different growth duration were significant.

**Table 4:** Cd accumulation at each growth stage in the main crop with different growth duration (kg ha^-1^).

Stage	Variety	Stem	Leaf	Grains spikelet	Total Cd accumulation
Transplanting stage to Full-Heading stage	XZX-45	744.0 ± 102.0c	113.0 ± 36.5c	70.8 ± 14.8c	927.8 ± 189.3c
	YLY-911	1190.0 ± 149.22b	210.0 ± 49.7b	157.0 ± 28.5b	1557.0 ± 319.6b
	HLY	1230.9 ± 189.73b	228.0 ± 42.9b	146.7 ± 21.7b	1605.6 ± 341.9b
	YY-4149	1708.0 ± 216.01a	439.2 ± 141.1a	288.0 ± 81.8a	2435.2 ± 583.2a
	YLY-9918	1965.0 ± 226.16a	457.6 ± 118.2a	303.0 ± 97.5a	2725.6 ± 457.1a
Full heading stage to Milky stage	XZX-45	1398.4 ± 189.3c	108.9 ± 14.8c	206.4 ± 21.9c	1713.7 ± 274.2c
	YLY-911	1918.8 ± 108.7b	143.7 ± 32.7b	278.0 ± 30.3b	2340.5 ± 179.7b
	HLY	1749.0 ± 192.8b	162.4 ± 27.5b	295.8 ± 37.2b	2207.2 ± 189.0b
	YY-4149	2135.0 ± 241.5a	243.6 ± 50.3a	393.6 ± 56.8a	2772.2 ± 222.5a
	YLY-9918	2117.4 ± 212.3a	230.0 ± 25.2a	371.0 ± 46.9a	2718.4 ± 151.9a
Milky stage to Maturity stage	XZX-45	1889.6 ± 278.8a	207.4 ± 47.1a	478.8 ± 98.4c	2575.8 ± 471.2a
	YLY-911	630.1 ± 191.3b	-84.2 ± 23.8b	801.0 ± 51.1b	1346.9 ± 193.9b
	HLY	811.8 ± 207.4b	-70.5 ± 20.9b	769.7 ± 69.6b	1511.0 ± 183.4b
	YY-4149	-651.0 ± 123.8c	-144.9 ± 31.2c	928.7 ± 67.8a	132.8 ± 24.6c
	YLY-9918	-795.6 ± 177.6c	-164.0 ± 35.8c	1152.8 ± 178.2a	193.2 ± 33.8c

Early-maturity variety: XZX-45, Xiangzaoxian 45; mid-maturity variety: YLY-911, Y Liangyou 911; HLY, Hengliangyou Jinnonngsimiao; late-maturity variety: YY-4149, Yongyou 4149; YLY-9918, Y Liangyou 9918. Significant differences (P< 0.05) among cultivars were determined by least significant difference tests and are indicated by different lowercase letters.

It was found that the Cd accumulation in each organ of each ratoon rice variety increased from the transplanting to the milky stage. In the milky to maturity stage, Cd accumulation in various grains spikelet varieties continued to increase, and as the growth duration got longer, the accumulation became greater. The Cd accumulation in the stems of early-maturity and mid-maturity varieties was a positive number, that is, the Cd accumulation amount was higher than the translocation amount, while the Cd accumulation in the stem of late-maturity varieties was a negative number, that is, the translocation amount was higher than the accumulation amount. Except for the early-maturity variety, the leaves of other varieties showed higher Cd translocation than accumulation.

**Table 5** shows that before the full-heading stage, the Cd accumulation in each organ among different varieties showed the trend of early-maturity > mid-maturity > late- maturity varieties. Among them, the Cd accumulation in the stem, stubble and leaf of the early-maturity variety was significantly higher than that of late-maturity varieties, and the Cd accumulation in grains spikelet varied significantly among varieties with different growth duration. During the full-heading to milky stage, the Cd accumulation in the stem and stubble of different varieties was in the order of late-maturity > mid-maturity > early-maturity varieties, and the difference was significant. The Cd accumulation in grains spikelet was highest in the early-maturity variety, and it was significantly higher than other varieties. During the milky to maturity stage, the Cd accumulation in grains spikelet was in the order of early-maturity > mid-maturity > late-maturity varieties, and the difference was significant. The Cd accumulation in other organs showed the order of late-maturity > mid-maturity > early-maturity varieties.

**Table 5:** Cd accumulation at each growth stage in the ratoon crop with different growth duration (kg ha^-1^).

Stage	Variety	Stem	Stubble	Leaf	Grains spikelet	Total Cd accumulation
Before the full-heading stage	XZX-45	2990.5 ± 338.4a	2507.5 ± 223.5a	319.0 ± 21.6a	332.0 ± 11.8a	6149.0 ± 261.2a
	YLY-911	2670.5 ± 161.2ab	2396.0 ± 149.9ab	316.8 ± 23.9a	288.0 ± 7.6b	5671.3 ± 201.7b
	HLY	2450.1 ± 209.4ab	2348.4 ± 124.7ab	317.1 ± 27.9a	289.6 ± 5.3b	5405.2 ± 271.8b
	YY-4149	2140.9 ± 121.9b	2051.6 ± 194.8b	258.0 ± 12.8b	254.0 ± 9.8c	4704.5 ± 333.2c
	YLY-9918	2279.1 ± 170.3b	2111.5 ± 152.7b	266.9 ± 17.4b	258. ± 10.1c	4915.5 ± 242.8c
Full heading stage to Milky stage	XZX-45	795.6 ± 83.5c	-942.8 ± 41.5c	106.0 ± 9.8a	288.0 ± 38.6a	246.8 ± 34.1c
	YLY-911	972.8 ± 77.9b	-739.0 ± 61.4b	91.4 ± 17.1a	216.0 ± 22.6b	541.2 ± 77.9b
	HLY	1016.1 ± 62.4b	-684.0 ± 62.9b	87.9 ± 13.4a	210.1 ± 37.7b	630.1 ± 121.4b
	YY-4149	1148.8 ± 58.6a	-507.9 ± 70.7a	100.2 ± 11.4a	208.8 ± 29.6b	950.0 ± 97.3a
	YLY-9918	1203.6 ± 121.3a	-559.6 ± 44.1a	119.5 ± 20.7a	217.8 ± 27.8b	981.3 ± 111.8a
Milky stage to Maturity stage	XZX-45	346.7 ± 39.1c	-883.8 ± 84.9c	159.8 ± 20.8c	659.7 ± 42.7a	282.4 ± 34.2c
	YLY-911	440.1 ± 46.9b	-675.8 ± 49.2b	272.0 ± 28.2b	549.4 ± 41.9b	585.7 ± 73.1b
	HLY	487.1 ± 38.5b	-717.3 ± 75.8b	285.4 ± 43.9b	576.1 ± 38.6b	631.3 ± 72.9b
	YY-4149	529.7 ± 45.9a	-551.0 ± 61.4a	433.8 ± 77.1a	395.0 ± 28.9c	807.5 ± 80.2a
	YLY-9918	594.3 ± 87.3a	-581.6 ± 40.6a	391.2 ± 67.2a	427.5 ± 56.1c	831.4 ± 97.8a

Early-maturity variety: XZX-45, Xiangzaoxian 45; mid-maturity variety: YLY-911, Y Liangyou 911; HLY, Hengliangyou Jinnonngsimiao; late-maturity variety: YY-4149, Yongyou 4149; YLY-9918, Y Liangyou 9918. Significant differences (P< 0.05) among cultivars were determined by least significant difference tests and are indicated by different lowercase letters.

Analyzing the dynamic patterns of Cd accumulation in various organs, except for stubble, Cd accumulation in ratoon rice with different growth duration continued to show an increasing trend throughout the growth stage. In terms of Cd accumulation in grains spikelet, when the growth duration was shorter the accumulation was greater. Cd accumulation in the stubble of various varieties showed that the accumulation amount was higher than the translocation amount during the transplanting to full-heading stage, while during the full-heading stage to maturity stage, the translocation amount was higher than the accumulation amount. When the growth period was shorter, the translocation amount was greater.

### 3.4 Differences in Cd translocation factors among ratoon rice varieties with different growth duration

The Cd translocation factors are an important indicator of the mutual translocation of Cd among different organs of rice plants. As shown in **Table 6**, the translocation factors of the root, stem, and leaf to grains spikelet at the full-heading and maturity stage among varieties with different growth duration all showed the trend of late-maturity > mid-maturity > early-maturity varieties, and the differences were significant. The translocation factors of root to stem and leaf at the full-heading and milky stage showed the trend of late-maturity > mid-maturity > early-maturity varieties. The differences among varieties with different growth duration at the full-heading stage reached a significant level, and the early-maturity variety at the milk-ripening stage was significantly lower than other varieties. The translocation factors of root to stem and leaf at the maturity stage were in the order of early-maturity > mid-maturity > late-maturity varieties, and the difference was significant.

**Table 6:** Cd translocation factors at each growth stage in the main crop of ratoon rice varieties with different growth duration.

Stage	Variety	Root to Grains spikelet	Stem to Grains spikelet	Leaf to Grains spikelet	Root to Stem	Root to Leaf
Full-Heading stage	XZX-45	0.039 ± 0.010c	0.240 ± 0.010c	0.600 ± 0.011c	0.158 ± 0.019c	0.062 ± 0.009c
	YLY-911	0.056 ± 0.006b	0.264 ± 0.013b	0.630 ± 0.018b	0.200 ± 0.012b	0.083 ± 0.007b
	HLY	0.052 ± 0.007b	0.267 ± 0.017b	0.647 ± 0.015b	0.192 ± 0.017b	0.085 ± 0.010b
	YY-4149	0.069 ± 0.007a	0.305 ± 0.020a	0.692 ± 0.030a	0.226 ± 0.013a	0.108 ± 0.011a
	YLY-9918	0.072 ± 0.011a	0.300 ± 0.010a	0.714 ± 0.037a	0.240 ± 0.022a	0.110 ± 0.013a
Milky stage	XZX-45	0.046 ± 0.009c	0.212 ± 0.006c	0.647 ± 0.026c	0.291 ± 0.019b	0.085 ± 0.007b
	YLY-911	0.066 ± 0.010b	0.224 ± 0.006b	0.750 ± 0.020b	0.345 ± 0.028a	0.108 ± 0.014a
	HLY	0.064 ± 0.009b	0.229 ± 0.009b	0.762 ± 0.016b	0.335 ± 0.020a	0.106 ± 0.011a
	YY-4149	0.090 ± 0.012a	0.248 ± 0.010a	0.800 ± 0.021a	0.328 ± 0.016a	0.108 ± 0.011a
	YLY-9918	0.090 ± 0.013a	0.247 ± 0.007a	0.814 ± 0.030a	0.323 ± 0.012a	0.103 ± 0.010a
Maturity stage	XZX-45	0.082 ± 0.009c	0.134 ± 0.030c	0.385 ± 0.047c	0.635 ± 0.034a	0.254 ± 0.010a
	YLY-911	0.108 ± 0.010b	0.194 ± 0.021b	0.588 ± 0.050b	0.572 ± 0.028b	0.194 ± 0.008b
	HLY	0.107 ± 0.013b	0.192 ± 0.037b	0.576 ± 0.059b	0.556 ± 0.036b	0.188 ± 0.009b
	YY-4149	0.155 ± 0.018a	0.288 ± 0.047a	0.743 ± 0.071a	0.430 ± 0.036c	0.167 ± 0.010c
	YLY-9918	0.162 ± 0.021a	0.325 ± 0.055a	0.800 ± 0.084a	0.467 ± 0.032c	0.170 ± 0.007c

Early-maturity variety: XZX-45, Xiangzaoxian 45; mid-maturity variety: YLY-911, Y Liangyou 911; HLY, Hengliangyou Jinnonngsimiao; late-maturity variety: YY-4149, Yongyou 4149; YLY-9918, Y Liangyou 9918. Significant differences (P< 0.05) among cultivars were determined by least significant difference tests and are indicated by different lowercase letters.

As shown in **Table 7**, in contrast to the main crop, the translocation factors of root, stubble, stem and leaf to grains spikelet at the full-heading and maturity stage among ratoon rice with different growth duration all showed the trend of early-maturity > mid-maturity > late-maturity varieties. Among them, the early-maturity variety at the full-heading and milk-ripening stage was significantly higher than late-maturity varieties, and the differences among varieties with different growth duration at maturity was significant. The translocation factors of root to stubble, stem and leaf at the full-heading stage were as follows: early-maturity > mid-maturity > late-maturity varieties. Among them, the early-maturity variety was significantly higher than late-maturity varieties. There were no obvious rules or differences in the translocation factors of the root to stubble, stem and leaf among varieties at the milky and maturity stage.

**Table 7:** Cd translocation factors at each growth stage in the ratoon crop of ratoon rice varieties with different growth duration.

Stage	Variety	Root to Grains spikelet	Stubble to Grains spikelet	Stem to Grains spikelet	Leaf to Grains spikelet	Root to Stubble	Root to Stem	Root to Leaf
Full-Heading stage	XZX-45	0.046 ± 0.007a	0.080 ± 0.003a	0.150 ± 0.009a	0.828 ± 0.027a	0.556 ± 0.033a	0.322 ± 0.36a	0.058 ± 0.006a
	YLY-911	0.042 ± 0.010ab	0.072 ± 0.003ab	0.146 ± 0.010ab	0.773 ± 0.028b	0.534 ± 0.037ab	0.282 ± 0.030ab	0.050 ± 0.008ab
	HLY	0.037 ± 0.007ab	0.071 ± 0.004ab	0.147 ± 0.011ab	0.762 ± 0.036b	0.514 ± 0.027ab	0.251 ± 0.024ab	0.045 ± 0.006b
	YY-4149	0.032 ± 0.005b	0.061 ± 0.005b	0.130 ± 0.010b	0.667 ± 0.033c	0.486 ± 0.025b	0.220 ± 0.025b	0.044 ± 0.007b
	YLY-9918	0.031 ± 0.005b	0.063 ± 0.004b	0.131 ± 0.009b	0.696 ± 0.042c	0.484 ± 0.028b	0.224 ± 0.022b	0.044 ± 0.006b
Milky stage	XZX-45	0.069 ± 0.007a	0.128 ± 0.005a	0.156 ± 0.006a	0.769 ± 0.049a	0.531 ± 0.045a	0.480 ± 0.050a	0.087 ± 0.015a
	YLY-911	0.062 ± 0.008ab	0.116 ± 0.006b	0.144 ± 0.005b	0.765 ± 0.044a	0.529 ± 0.031a	0.422 ± 0.031a	0.080 ± 0.020a
	HLY	0.063 ± 0.005ab	0.117 ± 0.005b	0.145 ± 0.005b	0.760 ± 0.038a	0.542 ± 0.041a	0.415 ± 0.028a	0.083 ± 0.0014a
	YY-4149	0.054 ± 0.005b	0.101 ± 0.007c	0.130 ± 0.007c	0.665 ± 0.046b	0.536 ± 0.038a	0.410 ± 0.035a	0.080 ± 0.010a
	YLY-9918	0.051 ± 0.007b	0.102 ± 0.008c	0.124 ± 0.008c	0.667 ± 0.040b	0.500 ± 0.041a	0.412 ± 0.029a	0.077 ± 0.017a
Maturity stage	XZX-45	0.115 ± 0.009a	0.187 ± 0.015a	0.156 ± 0.011a	0.646 ± 0.077a	0.595 ± 0.055a	0.817 ± 0.069a	0.172 ± 0.026a
	YLY-911	0.098 ± 0.007b	0.159 ± 0.009b	0.135 ± 0.009b	0.500 ± 0.052b	0.610 ± 0.061a	0.723 ± 0.052a	0.196 ± 0.028a
	HLY	0.089 ± 0.006b	0.162 ± 0.009b	0.127 ± 0.013b	0.500 ± 0.049b	0.551 ± 0.047a	0.700 ± 0.065a	0.178 ± 0.031a
	YY-4149	0.073 ± 0.009c	0.147 ± 0.009c	0.106 ± 0.007c	0.350 ± 0.055c	0.519 ± 0.054a	0.721 ± 0.05a	0.219 ± 0.031a
	YLY-9918	0.072 ± 0.009c	0.146 ± 0.009c	0.104 ± 0.010c	0.405 ± 0.040c	0.514 ± 0.045a	0.769 ± 0.044a	0.198 ± 0.035a

Early-maturity variety: XZX-45, Xiangzaoxian 45; mid-maturity variety: YLY-911, Y Liangyou 911; HLY, Hengliangyou Jinnonngsimiao; late-maturity variety: YY-4149, Yongyou 4149; YLY-9918, Y Liangyou 9918. Significant differences (P< 0.05) among cultivars were determined by least significant difference tests and are indicated by different lowercase letters.

### 3.5 Differences in daily average Cd accumulation rate of ratoon rice varieties with different growth duration

As shown in **Table 8**, in the main crop, the daily average Cd accumulation rate in various organs among varieties with different growth duration during the transplanting to full-heading stage showed a trend of late-maturity > mid-maturity > early-maturity varieties, and the differences were significant. There was no significant difference in the daily average Cd accumulation rate in various organs among varieties with different growth duration in the full-heading to milky stage. As for grains spikelet, the daily average Cd accumulation rate of late-maturity varieties was slightly higher, while that of the early-maturity variety was slightly lower. The daily average Cd accumulation rate in stem and leaf and the total Cd accumulation during the milk-ripening to maturity stage among varieties at different growth duration were in the order of early-maturity > mid-maturity > late-maturity varieties, and the difference was significant. However, the trend of grains spikelet was opposite (late-maturity > mid-maturity > early-maturity varieties), with the early-maturing variety being significantly lower than other varieties.

**Table 8:** Daily average Cd accumulation rate in each organ during the main crop of ratoon rice varieties with different growth duration (mg ha^-1^·d).

Stage	Variety	Stem	Leaf	Grains spikelet	Total Cd accumulation rate
Transplanting stage to Full-Heading stage	XZX-45	8.9 ± 0.9c	1.3 ± 0.3c	0.8 ± 0.2c	11.0 ± 2.1c
	YLY-911	13.5 ± 1.4b	2.4 ± 0.6b	1.8 ± 0.3b	17.7 ± 3.0b
	HLY	14.0 ± 1.8b	2.6 ± 0.5b	1.7 ± 0.4b	18.2 ± 3.7b
	YY-4149	18.6 ± 1.5a	4.8 ± 0.9a	3.1 ± 0.5a	26.5 ± 3.5a
	YLY-9918	21.4 ± 2.2a	5.0 ± 0.7a	3.3 ± 0.7a	29.6 ± 4.2a
Full heading stage to Milky stage	XZX-45	139.8 ± 21.5a	10.9 ± 1.3a	19.9 ± 2.7a	171.4 ± 30.2a
	YLY-911	137.1 ± 28.2a	10.3 ± 1.7a	20.1 ± 2.3a	167.2 ± 29.5a
	HLY	124.9 ± 14.6a	11.6 ± 2.0a	21.1 ± 3.0a	157.7 ± 27.1a
	YY-4149	125.6 ± 21.3a	14.3 ± 2.1a	23.2 ± 3.2a	163.1 ± 31.7a
	YLY-9918	124.6 ± 17.8a	13.5 ± 2.5a	22.8 ± 1.9a	159.9 ± 31.4a
Milky stage to Maturity stage	XZX-45	145.4 ± 34.7a	16.0 ± 2.9a	36.8 ± 2.9c	198.1 ± 39.0a
	YLY-911	35.0 ± 3.6b	-4.7 ± 0.9b	42.5 ± 2.0b	74.8 ± 17.9b
	HLY	45.1 ± 5.1b	-3.9 ± 1.1b	42.8 ± 3.8b	83.9 ± 21.3b
	YY-4149	-29.6 ± 3.0c	-6.6 ± 0.9c	44.2 ± 3.1b	6.0 ± 0.6c
	YLY-9918	-36.2 ± 4.8c	-7.5 ± 1.5c	52.4 ± 5.1a	8.8 ± 1.0c

Early-maturity variety: XZX-45, Xiangzaoxian 45; mid-maturity variety: YLY-911, Y Liangyou 911; HLY, Hengliangyou Jinnonngsimiao; late-maturity variety: YY-4149, Yongyou 4149; YLY-9918, Y Liangyou 9918. Significant differences (P< 0.05) among cultivars were determined by least significant difference tests and are indicated by different lowercase letters.

It was found that the daily average Cd accumulation rate of grains spikelet varieties was highest during the milky to maturity stage. Except for the early-maturity variety, the Cd accumulation rate in the stem and leaf and the total Cd accumulation of other varieties were highest during the full-heading to milky stage, while the early-maturity variety was highest during the milky to maturity stage, but there was no significant difference among this and the full-heading to milky stage.

As shown in **Table 9**, the daily average Cd accumulation rate in various organs among varieties with different growth duration during the transplanting to full-heading stages of the ratoon crop was as follows: early-maturity > mid-maturity > late-maturity varieties. Among them, except for leaf, the differences in the daily average Cd accumulation rate among varieties with different growth duration in other organs was significant. There was no significant difference in stem daily average Cd accumulation rate among varieties with different growth duration during the full-heading to milky stage; The daily average Cd accumulation rate of stubble and total Cd accumulation rate showed the trend of late-maturity > mid-maturity > early-maturity varieties, and the differences were significant; The daily average Cd accumulation rate in leaf and grains spikelet showed the trend of early-maturity > mid-maturity > late-maturity varieties. Among them, the accumulation in the leaf in the early-maturity variety was significantly higher than other varieties, and grains spikelet showed significant differences among varieties with different growth duration. There was no significant difference in stem daily average Cd accumulation rate among varieties during the milky to maturity stage. The daily average Cd accumulation rate in stubble, leaf and total Cd accumulation was in the order of late-maturity > mid-maturity > early-maturity varieties, among which the late-maturity varieties were significantly higher than the early-maturity variety; The daily average Cd accumulation rate of grains spikelet was in the order of early-maturity > mid-maturity > late-maturity varieties, and the difference was significant.

**Table 9:** Average daily Cd accumulation rate in each organ during the ratoon crop of ratoon rice varieties with different growth duration (mg ha^-1^·d).

Stage	Variety	Stem	Stubble	Leaf	Grains spikelet	Total Cd Accumulation Rate
Before the full-heading stage	XZX-45	69.5 ± 5.3a	58.3 ± 2.9a	7.4 ± 0.8a	7.7 ± 0.7a	145.0 ± 10.7a
	YLY-911	59.3 ± 3.9b	53.2 ± 1.5b	7.0 ± 0.7a	6.4 ± 0.6b	126.0 ± 6.2b
	HLY	54.4 ± 4.0b	52.2 ± 1.9b	7.0 ± 0.8a	6.4 ± 0.5b	120.1 ± 7.4b
	YY-4149	44.6 ± 3.4c	42.7 ± 2.0c	5.3 ± 0.5b	5.3 ± 0.4c	98.0 ± 4.9c
	YLY-9918	47.2 ± 2.8c	44.0 ± 2.5c	5.5 ± 0.5b	5.4 ± 0.4c	102.4 ± 6.8c
Full heading stage to Milky stage	XZX-45	72.3 ± 5.0a	-85.7 ± 7.2c	9.6 ± 1.7a	26.2 ± 4.1a	22.4 ± 2.0c
	YLY-911	69.5 ± 3.3a	-52.8 ± 7.0b	6.5 ± 1.3b	15.4 ± 2.2b	38.7 ± 4.1b
	HLY	72.6 ± 5.8a	-48.9 ± 6.6b	6.3 ± 1.0b	15.0 ± 2.5b	45.0 ± 3.8b
	YY-4149	63.8 ± 3.6a	-28.2 ± 3.2a	5.6 ± 0.8b	11.6 ± 1.2c	52.8 ± 3.2a
	YLY-9918	66.9 ± 4.8a	-31.1 ± 4.8a	6.6 ± 1.0b	12.0 ± 1.0c	54.5 ± 5.5a
Milky stage to Maturity stage	XZX-45	23.1 ± 1.4a	-58.9 ± 4.8c	10.7 ± 1.4b	44.0 ± 4.1a	18.8 ± 2.0b
	YLY-911	22.2 ± 2.0a	-33.8 ± 3.0b	13.6 ± 1.2b	27.5 ± 3.0b	29.3 ± 3.6a
	HLY	24.4 ± 1.8a	-35.9 ± 4.4b	14.3 ± 1.0b	28.8 ± 3.7b	31.6 ± 4.1a
	YY-4149	23.0 ± 1.2a	-24.0 ± 2.7a	18.9 ± 2.4a	17.2 ± 2.2c	35.1 ± 3.3a
	YLY-9918	25.8 ± 2.6a	-25.3 ± 3.2a	17.0 ± 1.8a	18.6 ± 2.6c	36.1 ± 3.7a

Early-maturity variety: XZX-45, Xiangzaoxian 45; mid-maturity variety: YLY-911, Y Liangyou 911; HLY, Hengliangyou Jinnonngsimiao; late-maturity variety: YY-4149, Yongyou 4149; YLY-9918, Y Liangyou 9918. Significant differences (P< 0.05) among cultivars were determined by least significant difference tests and are indicated by different lowercase letters.

It was found that the daily average Cd accumulation rate patterns in various organs of the ratoon crop were relatively consistent among varieties. The daily average Cd accumulation rate in stubble and the total Cd accumulation in each rice variety was highest during the transplanting to full-heading stage, the daily average Cd accumulation rate in the stem was the highest during the full-heading to milky stage, and the Cd accumulation rate in the leaf and grains spikelet was the highest during the milky to maturity stage.

## 4 Discussion

The differences in the traits among different rice varieties are affected by the genetic characteristics of the rice varieties themselves, including growth duration (Ping et al., 2018). The length of the growth duration is one of the key factors affecting crop growth, and previous studies have been conducted on this (Wang et al., 2019; Ye et al., 2019; Shrestha et al., 2022). Research showed that if growth duration of rice was short, the vegetative growth duration was shortened, resulting in insufficient accumulation of nutrients in the early stage (Ye et al., 2019). However, if the growth duration of late rice was too long, the delay in heading stage will cause the daily average temperature and effective accumulated temperature in the filling stage to decrease, thus affecting the grain filling speed and time (Shrestha et al., 2022).

The current study focused on ratoon rice and found that the dry matter weight of grains spikelet at each growth stage in the main crop showed a significant increasing trend with the extension of the growth duration, but there was no difference in the dry matter weight of stem and leaf at the maturity stage. This may be because the temperature was higher during the main crop’s maturity stage. Longer growth duration required more time for the stem and leaf to translocation nutrients to the grains spikelet. Therefore, the dry matter distribution ratio of the stem and leaf decreased, but it increased in the grains spikelet. Varieties with short growth duration had less accumulation of nutrients in the above ground matter in the early stage and needed a shorter time to translocation nutrients to the grains spikelet, thus the dry matter distribution ratio of stem and leaf was relatively higher. Plants in the ratoon crop were grown and headed from the dormant buds on the rice stubble of the main crop. Therefore, part of their nutrients in the early stage came from the absorption of the old roots in the main crop and the accumulation and translocation of rice stubble, and the plant then relied on new roots to absorb nutrients (Wu et al., 2023). In the current study, the dry matter weight of organs in each growth stage of the ratoon crop generally increased with the extension of the growth duration of the varieties, and the dry matter weight of grains spikelet among varieties with different growth duration was significantly different. It can be seen that the short growth duration of rice results in reducing plant growth. The growth of ratoon rice, especially in the ratoon crop, mainly depends on tillering ability and regeneration ability. The stronger the tillering ability, the greater the rice growth (Torres et al., 2020). Study had shown that shortening the vegetative growth stage of rice will affect rice tillering and reduce the rice growth amount. The shortened reproductive growth stage will affect the formation of spikelets. Coupled with the reduced accumulation of nutrients in the early stage, and the weak single stem at the heading stage, this will aggravate the degradation of spikelets, which is not conducive to the accumulation of substances in the panicle (Guo et al., 2022).

There are currently no studies on the differences in Cd translocation and accumulation in rice during different growth duration, but most studies have shown that varieties with a long growth duration accumulate more nutrients and absorb more nitrogen (Chen et al., 2017). Safdar et al. (2008) studied three rice varieties with different growth types and found that the total nitrogen uptake of varieties with a long growth duration was significantly greater than that of varieties with a short growth duration and was also more stable from year to year. Decouard et al (Decouard et al., 2022). also reported that the total nitrogen absorption of varieties with a long growth duration was greater than that of varieties with a medium growth duration, and this did not change with differences in soil fertility levels. The plants had a similar performance with five different soil fertility levels.

The current study focused on the main crop of ratoon rice and found that with the extension of the growth duration, the Cd concentration of grains spikelet increased in the main crop, and the Cd accumulation at each growth stage increased significantly. Analysis of the mechanism revealed that the Cd translocation factors from root, stem and leaf to grains spikelet increased significantly with the extension of the growth duration. At the same time, the daily average Cd accumulation rate from the transplanting to full-heading stage and the milky to maturity stage was also consistent with the trend of Cd accumulation, which meant that a longer growth duration led to a higher daily average Cd accumulation rate.

According to the patterns of Cd concentration and accumulation in the aboveground parts (stem, leaf), it was found that as the growth duration extended, the Cd concentration in each part at the full-heading stage and milk-ripening stage and the Cd accumulation in each part during the transplanting to milky stage gradually increased. However, the opposite trend was shown at the maturity stage, and the Cd translocation amount in the stem and leaf of late-maturity varieties and in the leaf of mid-maturity varieties during the milky to maturity stage was higher than the Cd accumulation amount. Research showed that the mid-filling to maturity stage was the most important stage for Cd accumulation in grains spikelet during the main crop (Taek et al., 2010). At the same time, the source of Cd in the rice grains spikelet, especially in the later stage, mainly came from the translocation to other aboveground organs. Therefore, when the milky to maturity stage was longer, it might take longer for nutrients to be translocationed from the aboveground organs to the grains. At the same time, the Cd element will be translocationed along with the nutrients, thus resulting in a larger Cd translocation factor. In addition, the higher temperature during the milky to maturity stage in the main crop may further promote the translocation of Cd in the aboveground parts to grains spikelet. Therefore, the distribution ratio of Cd in stem and leaf was reduced, resulting in an increase in Cd concentration and accumulation in grains spikelet. In addition, since there was no brown rice at the full-heading and milky stages, grain spikelets were measured at these two growth stages. In order to unify the samples at each growth stage, the Cd concentration of the ratoon rice sample parts, which are still grains spikelets, was measured at the maturity stage. Study had shown that there was no significant difference in Cd concentration in rice grains spikelets and brown rice. Because the Cd translocation factors from other parts of rice (roots, stem and leaf) to grain spikelets is roughly the same as brown rice (Yang et al., 2020).

In contrast to the main crop, with the extension of the growth duration, the Cd concentration and accumulation of grains spikelet in the ratoon crop gradually decreased, and the differences among varieties with different growth duration reached significant levels. It was found that the Cd translocation factors from root, stubble, stem, and leaf to grains spikelet at each growth stage decreased significantly with the extension of the growth duration. The daily average Cd accumulation rate of grains spikelet was also highest in the early-maturity variety and lowest in late-maturity varieties. The Cd concentration in the aboveground parts (stubble, stem, leaf) at each growth stage also decreased with the extension of the growth duration. The Cd translocation factors from root to stubble, stem and leaf at the full-heading stage was significantly higher in the early-maturity variety than in late-maturity varieties. There was no obvious difference in the overall root to above-ground parts Cd translocation factors among varieties during the milky stage and maturity stage. It can be seen that the Cd translocation factors among varieties with different growth duration during the ratoon crop did not have a great impact on the Cd concentration in various organs. The Cd accumulation among varieties with different growth duration was inconsistent with the trend of Cd concentration at each growth stage. The order of early-maturity > mid-maturity > late-maturity varieties at the full-heading stage was followed, while the trend of Cd accumulation at the milk-ripening stage and the maturity stage was the opposite.

The stubble left behind by the main crop of ratoon rice form the material source for the ratoon crop, and the output of nutrients in the stubble has a decisive impact on axillary bud germination and growth (Huang et al., 2023). Therefore, while the residual nutrients in the stubble are rapidly transferred to the ratoon tillers, the Cd accumulated in the main crop may be transferred to the ratoon seedlings with the nutrients, causing a greater impact on the Cd concentration of the plants in the ratoon crop. In the current study, the Cd concentration in the aboveground parts (root, stem, and leaf) at maturity of the main crop was higher in the early-maturity variety and lower in the late-maturity varieties, and the difference in Cd concentration in stem and leaf was significant. At the same time, studies have shown that the more Cd absorbed and accumulated by rice, the higher the intensity of Cd translocation from the root to the shoot and the higher the translocation efficiency of Cd from the shoot to the grain (Zhang et al., 2017). Therefore, the Cd concentration in organs at each growth stage of the ratoon crop was higher in the early-maturity variety and lower in the late-maturity varieties. Increasing plant Cd concentration or increasing dry matter weight, or both, can significantly increase plant Cd accumulation (Uraguchi and Fujiwara, 2012). The Cd accumulation in the aboveground parts (stubble, stem, leaf) before the full-heading stage was higher in the early-maturity variety, which may be due to its higher Cd concentration. The Cd accumulation during the full-heading to maturity stage was higher in late-maturity varieties, which may be due to their higher dry matter weight. However, the trend of Cd accumulation in grains spikelet was consistent with the Cd concentration in grains spikelet. It can be seen that the main way to reduce the accumulation of Cd in ratoon rice grains is to control its Cd concentration.

The growth process of rice varieties with different growth duration is inconsistent, and the temperature and light resources they utilize are also different, such as rainfall, temperature, and light hours. In the rice areas of southern China, the growth cycle of ratoon rice from the main crop to the ratoon crop is relatively long, approximately from March to October. During this growing season, the temperature change trend shows an increase first and then a decrease, with a temperature change range of 20-25°C (Ma et al., 2022). Research showed that rice requires specific temperatures during development stages such as tillering, heading and maturity (Guo-cai et al., 2014). Temperature is also an important factor affecting Cd accumulation and translocation in rice. Keqiang et al (Keqiang et al., 2019). believed that warming increased the number of fine roots (diameter ≤0.5 mm), expanded root surface active sites, and promoted the absorption of heavy metals by roots. At the same time, warming increased the transpiration of leaf and promoted the flow of xylem from the nutrient solution to the upper organs, thereby promoting the translocation of heavy metals. Different growth duration among varieties will lead to different temperatures during rice growth, thereby affecting the accumulation and translocation of Cd in rice.

In the current study, the active accumulated temperature and effective accumulated temperature in the main crop were consistent with the trend of Cd concentration and accumulation in grains spikelet (**Table 3**; **Supplementary Figure S2**) and showed an increasing trend with the extension of the growth duration of the variety. The daily average temperature, daily maximum temperature, and daily minimum temperature during the sowing to full-heading stage and the milky to maturity stage were all highest in late-maturity varieties, that is, the longer the growth duration of the varieties, the higher the accumulated temperature and daily average temperature during the whole period, and the Cd concentration and accumulation in grains spikelet will increase accordingly. The daily average temperature, daily maximum temperature and daily minimum temperature at each growth stage of the ratoon crop were consistent with the trend of Cd concentration and accumulation in grains spikelet and showed a decreasing trend with the extension of the growth duration (**Table 3**). Liu et al (Liu et al., 2021). believed that the tillering to full-heading stage of rice was the most important for Cd accumulation in rice, while the filling to maturity stage was the key stage to control the translocation of Cd to the grains. A certain degree of high temperature can promote the synthesis and accumulation of assimilates in rice during the vegetative growth stage, while the transpiration of rice in high temperatures in the mid-filling to maturity stage will enhance the absorption and translocation of Cd. It can be seen that although the temperature in the early stage of the main crop of ratoon rice was low, as the growth duration extended, the temperature gradually increased, therefore, late-maturity varieties had higher Cd absorption and translocation efficiency during the milky to maturity stage, and grains spikelet had higher Cd concentration and accumulation. The temperature was higher in the early part of the ratoon crop, but as the growth duration extended, the temperature gradually decreased. Therefore, the early-maturity variety was in the high-temperature period from the milky to maturity stage, the Cd translocation rate in the above-ground part of grains spikelet was higher, and the Cd accumulation amount was large. However, the temperature during the milky to maturity stage of late-maturity varieties was lower than that of the early-maturity variety, thus, the Cd transfer rate to grains spikelet was relatively low.

In this study, only one or two varieties of each growth duration type of ratoon rice were selected for testing. Considering that soil Cd levels in different regions and different annual temperature and light conditions also have different effects on the absorption, accumulation and translocation of Cd in rice plants, and there is also the possibility of differences among different varieties in the same growth duration, multi-variety, multi-region and multi-year research should be conducted on the screening of ratoon rice varieties with different growth duration. At the same time, the differential mechanisms should be further summarized in terms of physiology and molecular biology to provide scientific support for the safe and stable development of ratoon rice.

## 5 Conclusion

With the extension of the growth duration, the Cd concentration and accumulation of grains spikelet showed an increasing trend in the main crop, and a decreasing trend in the ratoon crop. The main reason was that the root, stubble, stem, leaf to grains spikelet Cd translocation factors and the daily average Cd accumulation rate of grains spikelet were relatively high. Combined with climatic conditions, it was found that the daily average temperature and the accumulated temperature of ratoon rice were consistent with the trend of Cd concentration and accumulation in grains spikelet. Therefore, appropriately shortening the growth duration of the main crop and appropriately extending the growth duration of the ratoon crop are important ways to reduce Cd accumulation in ratoon rice.

## Data availability statement

The original contributions presented in the study are included in the article/**Supplementary Material**. Further inquiries can be directed to the corresponding authors.

## Ethics statement

The author states that the rice involved in this study do not involve ethical relations. Experimental research on plants, including the collection of plant material, complies with relevant institutional, national, and international guidelines and legislation.

## Author contributions

SY: Data curation, Formal analysis, Methodology, Writing – review & editing. YJ: Data curation, Formal analysis, Writing – original draft. PC: Methodology, Writing – review & editing. NT: Funding acquisition, Project administration, Resources, Writing – review & editing. WZ: Funding acquisition, Supervision, Writing – review & editing. ZY: Funding acquisition, Project administration, Supervision, Writing – review & editing.
