# Supplementary material for: Difference in Cd accumulation among varieties with different growth duration corresponding to typical agro-climate condition in rice ratooning system
Source: Front Plant Sci. 2024 May 8;15:1383428. doi: 10.3389/fpls.2024.1383428 (PMC11109407; doi:10.3389/fpls.2024.1383428)
Supplement: Supplementary file 1 [file Image_1.pdf]

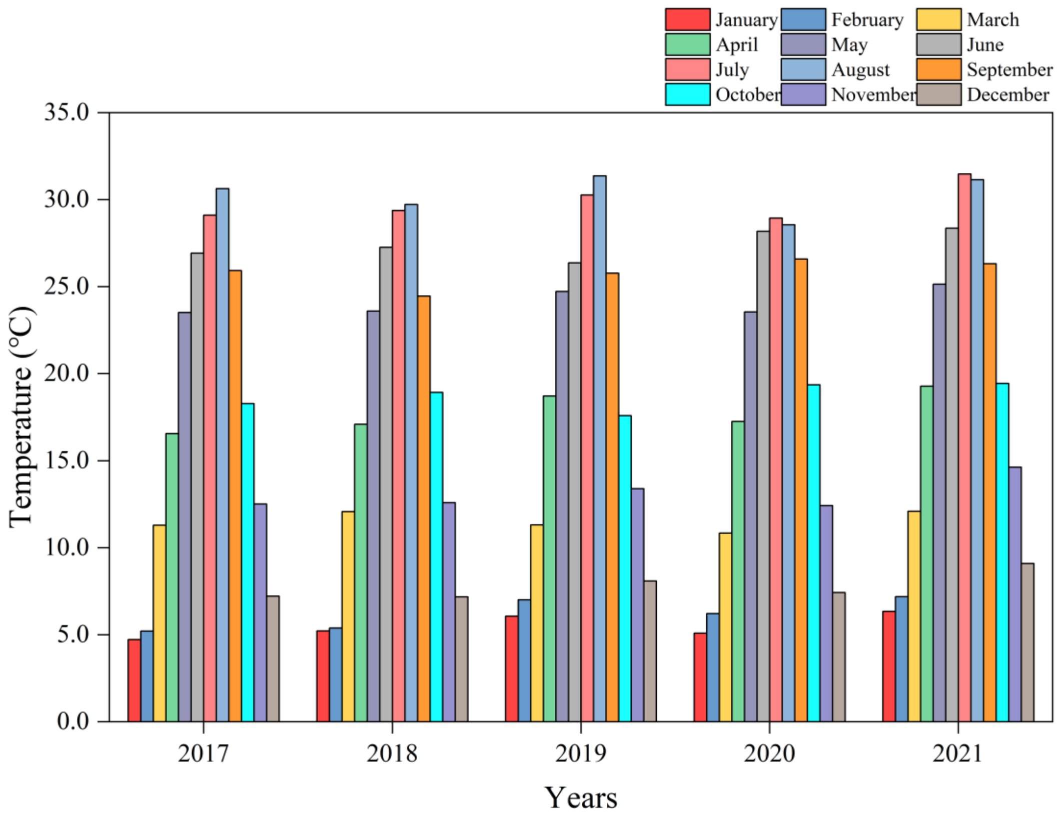

Fig. S1. Average temperatures in each month from 2017 to 2021.

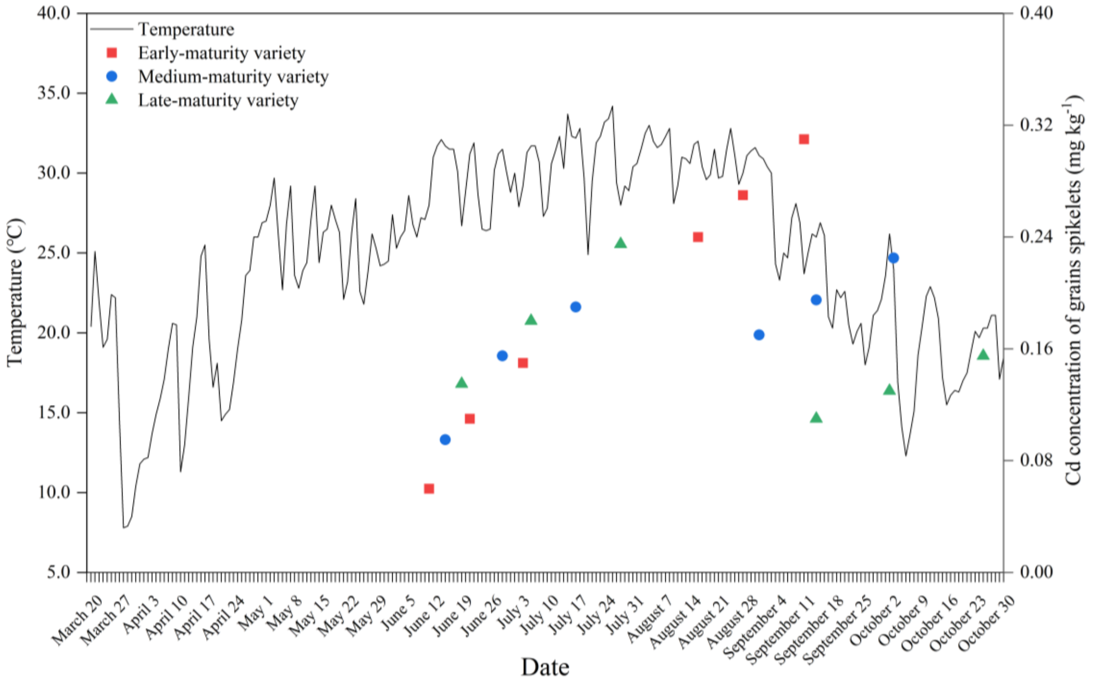

Fig. S2. Trends in Cd concentration and temperature in grain spikelets of ratoon rice varieties at different growth duration. For each variety, the growth stages are full-heading, milky, maturity in main crop, and full-heading, milky, maturity in ratoon crop as time goes by.
